# Impact of co-administration of apricot kernels and caffeine on adult male diabetic albino rats

**DOI:** 10.3389/fphys.2024.1358177

**Published:** 2024-11-14

**Authors:** Ahmed El Sayed Nour El-Deen, Ahmad Mohamad Taha, Almoatazbellah Elsayed, Ahmed Noaman Ali, Reda Samir Taha

**Affiliations:** ^1^ Department of Basic Medical and Dental Sciences, Faculty of Dentistry, Zarqa University, Zarqa, Jordan; ^2^ Department of Physiology, Faculty of Medicine, Al-Azhar University, Assiut, Egypt; ^3^ Department of Physiology, Faculty of Medicine, Port Said University, Port Said, Egypt; ^4^ Department of Pathology, Faculty of Medicine, Al-Azhar University, Assiut, Egypt; ^5^ Department of Oral Pathology, Faculty of Dentistry, Tanta University, Tanta, Egypt; ^6^ Department of Anatomy, Faculty of Medicine in Damietta, Al-Azhar University, Assiut, Egypt

**Keywords:** type 2 diabetes mellitus, apricot kernels, caffeine, Antioxidant, nutritarian

## Abstract

The purpose of this study is to evaluate the impacts of apricot kernels and caffeine on blood glucose, lipid profile, insulin secretion, and antioxidant effect in diabetic rats. Forty adult male albino rats were divided into five groups: normal control, diabetic control, diabetic rats treated with apricot kernels, diabetic rats treated with caffeine, and diabetic rats treated with apricot kernels plus caffeine. Fasting samples were collected at the end of the study for analysis, and pieces of liver and pancreatic tissues were removed for histological analysis. There was a significant decrease in blood glucose, glycated hemoglobin, body weight, total cholesterol, triglyceride, and low-density lipoprotein cholesterol (LDL-C) levels and a significant increase in insulin and high-density lipoprotein cholesterol (HDL-C) levels in the kernel and caffeine-treated groups. However, there was little histological alteration in the liver or pancreas, and no significant differences were observed in the histological findings between groups. Overall, it can be concluded that apricot kernel and caffeine had a positive effect in decreasing blood glucose and harmful lipid profile and that caffeine had a synergistic effect on the apricot kernel.

## 1 Introduction

Diabetes mellitus (DM) is a fast-growing health problem that causes significant economic burden worldwide, especially the adult-onset or type 2 diabetes mellitus (T2DM) (Arokiasamy et al., 2021). It was reported that the global prevalence of diabetes among adults has reached alarming levels all over the world; it was nearly 9.4% in the year 2019, which indicated approximately 462 million adults had DM worldwide, and this number would increase to 700 million in 2045 (Alp et al., 2023).

The increased incidence of known risk factors for T2DM, such as physical inactivity, obesity, and unhealthy diet, increased the morbidity and mortality of the world population (Arokiasamy et al., 2021).

In the early stages of T2DM, oxidative stress, together with other factors, upregulated the activation of AMP-activated protein kinase (AMPK) and enhanced pancreatic beta cell survival and function. However, in chronic cases, chronic oxidative stress conditions can be the result of the continuous production of reactive oxygen species that chronically activate AMPK and result in damage to pancreatic beta cells with repressed insulin release (Fu et al., 2013). Therefore, reducing inflammation and oxidative stress can be one of the main therapeutic objectives for managing T2DM (Vivó-Barrachina et al., 2022). Seeds and nuts can be useful sources for the aforementioned therapeutic targets for T2DM. Their bioactive components have high nutraceutical and medicinal value (Shalaby et al., 2015; El-Deen et al., 2018; El-Deen et al., 2019; El-Deen et al., 2024). They proved to be beneficial in controlling body weight, blood glucose levels, and blood pressure, along with reduction of coronary heart disease and levels of blood cholesterol and triacylglycerol (Sater et al., 2016; Shalaby et al., 2016; El-Deen et al., 2018; Jugran et al., 2021).

Apricot (*Prunus armeniaca* L.) is a fruit that belongs to the genus *Prunus* of the Rosaceae family. The fruits contain a kernel enclosed in a stone seed. The kernel can be a food by-product for its high content of dietary protein (14%–45%), oil (28%–66.7%), and phytochemicals such as phenolic compounds, carotene, phytosterols, and tocopherol (Mohamed et al., 2021). Apricot kernel contains caffeic acid and gallic acid, which are used in anti-asthmatic, antiseptic, sedative, emetic, and laxative medicinal preparations. It can also be beneficial in the treatment of various diseases such as cardiovascular disease and breast cancer (Akhone et al., 2022; Hamid et al., 2022). They can also be used in developing a source of antioxidant biocompounds and some hypoglycemic agents like anthocyanin (Akbari et al., 2022).

Caffeine (1,3,7-trimethylxanthine) is present in different sources, mainly coffee, tea, and soft drinks (Heckman et al., 2010). Coffee contains several bioactive compounds that are well known for being antioxidants (Cämmerer and Kroh, 2006; Neves et al., 2018). Antioxidants have been shown to reduce diabetes complications by improving cell function in animal models, suggesting that enhancing antioxidant defense mechanisms in pancreatic islets may be a valuable pharmacologic approach to managing diabetes. Several studies have demonstrated that a higher consumption of coffee is associated with a lower risk of developing T2DM (Huxley et al., 2009; Muley et al., 2012; Bidel and Tuomilehto, 2013).

Our hypothesis is that apricot kernel can reduce the blood glucose concentration in type 2 diabetic patients and may also reduce complications resulting from chronic uncontrolled diabetes because it contains antioxidants and some hypoglycemic agents. So, the aim of this work was to study the effect of apricot kernel with or without caffeine on blood glucose concentration and lipid and antioxidant profiles in diabetic rats.

## 2 Materials and methods

### 2.1 Preparation of apricot kernel and caffeine

Apricot kernels were collected from an Egyptian local market during the fruit-ripening season. Fruits were washed thoroughly under running tap water, and then kernels were removed from the fruits; the kernels were dried in an air oven at 50°C for 24 h and ground to obtain fine powder using an air mill according to the method described by Musa and Sciences (2010). Then, the powder was stored in dark glass bottles at normal room temperature until further use. Caffeine (1,3,7-trimethylxanthine) was obtained from local Egyptian markets.

### 2.2 Experimental animals

Forty adult male albino rats of the local strain weighing 150 ± 20 g were purchased from the animal house of Assiut University. All animal procedures were conducted in strict conformity with the Ethical Committee Guidelines for the Care and Use of Laboratory Animals of the Faculty of Medicine, Al-Azhar University.

Animals were kept in suitable cages (20 × 32 × 20 cm for every 3 rats) in an environmentally controlled breeding room (temperature: 22°C ± 2°C, humidity: 60% ± 5%, 12 h dark/light cycle); the animals were maintained on a standard diet of commercial rat food formula (El-Nasr-Pharmaceutical Co., Cairo, Egypt) and tap water.

T2DM was induced in male albino rats fed with a high-fat diet for 3 weeks, followed by intraperitoneal injection of a low-dose streptozotocin (40 mg/kg/body weight daily for five successive days) from the 22nd day of treatment with a high-fat diet (Akbarzadeh et al., 2007). Rats with fasting blood glucose level (FBG) > 200 mg/dL were selected as diabetic and were included in this study (Furman, 2015). Blood glucose levels were estimated.

### 2.3 Experimental design

All procedures were conducted at Al-Azhar University, Assuit, Egypt. Rats were weighed at the beginning and the end of the experiment.

Animals were randomly divided into 5 groups (*n* = 8) as follows:

(Group1) Normal control rat treated with normal saline: received daily 1 mL/kg of sterile saline by gavage for 1 month.
(Group 2) Type 2 diabetic control rats treated with normal saline, received daily 1 mL/kg of sterile saline by gavage for 1 month.
(Group 3) Type 2 diabetic rats treated with apricot kernels as powder, received daily as 5% of the diet for 1 month.
(Group 4) Type 2 diabetic rats treated with caffeine, received daily 20 mg/kg by gavage.
(Group 5) Type 2 diabetic rats treated with apricot kernels as powder, received daily as 5% of the diet plus received daily 20 mg/kg by gavage.

### 2.4 Blood sampling

After 30 days of treatment, the rats were sacrificed by cervical dislocation. Blood was immediately collected from the aorta into a dry clean glass heparinized centrifuge tube and centrifuged for 15 min at 5,000 rpm to separate the plasma, which was carefully aspirated and transferred into a clean cuvette tube and stored in a deep freezer for further biochemical assays.

### 2.5 Biochemical analysis

#### 2.5.1 Glycated hemoglobin (HbA1c)

HbA1c was measured with the help of BioRad D10-HbA1c analyzer, CAL-REMEDIES (Shoaib et al., 2020).

#### 2.5.2 Lipid profile

Total cholesterol (TC), high-density lipoprotein cholesterol (HDL-C), low-density lipoprotein cholesterol (LDL-C), and triglycerides (TG) were measured in sera using enzymatic colorimetric kits (Stanbio Laboratory, United States, and ELITech Group, France) according to Tietz (1990).

#### 2.5.3 Insulin level

The levels of insulin in plasma were estimated by a commercial rat enzyme-linked immunosorbent assay kit (Abnova, Germany) according to the enclosed manufacturer’s protocol.

#### 2.5.4 Antioxidant profile analysis

Lipid peroxidation was estimated by measuring malondialdehyde (MDA) levels according to the method described (Ohkawa et al., 1979). Using commercially available kits from Biodiagnostic Chemical Company (Cairo, Egypt), glutathione peroxidase (GPx) activity was estimated according to Rotruck et al. (1973). To estimate the rate of glutathione oxidation by H_2_O_2_, one unit of GPx activity per minute is defined as the amount of enzyme needed for the conversion of 1 μmol of reduced glutathione to its oxidized form. Catalase (CAT) activity was estimated in the plasma according to the method described by Aebi (1984) (HADWAN et al., 2018) using commercially available kits from Biodiagnostic Chemical Company (Cairo, Egypt).

### 2.6 Histopathology

At the end of the experiment, after scarification, a small piece of liver and pancreatic tissue were removed for histological analysis. The tissues were fixed in 10% formalin (diluted to 10% with normal saline). The tissues were dehydrated in graded concentrations of ethyl alcohol and cleared with xylene, embedded in molten paraffin wax, and sectioned at 5 µ. Tissue sections were fixed on glass slides and stained with hematoxylin and eosin for light microscopy (Kilari et al., 2020).

The histological changes were evaluated in nonconsecutive, randomly chosen × 200 histological fields using a light microscope (the Nikon Eclipse E200 student microscope).

### 2.7 Statistical analysis

We used R software version 4.1.1 (Packages: tidyverse, ggpubr, and rstatix) to perform biomedical analysis. We applied one-way ANOVA followed by Tukey’s post hoc test to compare different parameters. Mean ± SD described our results. *p*-value < 0.05) was considered a statistically significant value.

## 3 Results

### 3.1 Kernels and caffeine decreased blood glucose level in diabetic rats

**FIGURE 1:**
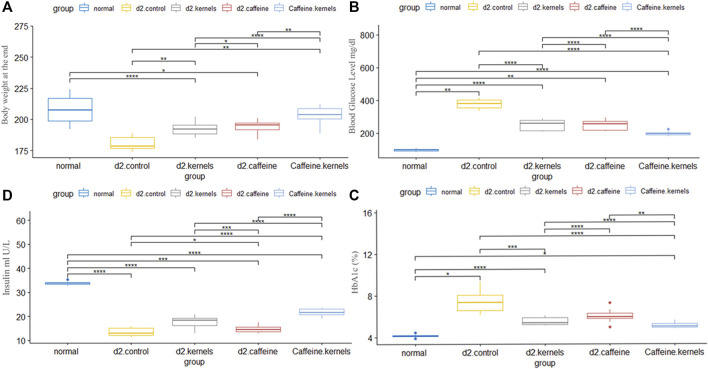
Administration of apricot kernel and caffeine improved blood glucose level and related factors in diabetic rats. **(A)** Body weight differences between groups at the end of the experiment. **(B)** Glucose concentration. **(C)** HbA1c level in different groups. **(D)** Insulin level in diabetic rats after different treatments. Healthy control rats (normal), control diabetic rats (d2.control), diabetic rats treated with apricot kernel (d2.kernels), diabetic rats treated with caffeine (d2.caffeine), and diabetic rats treated with both caffeine and apricot kernel (Caffeine.kernels). One-way ANOVA and *post hoc* test, *p*-value* <0.05, **<0.01, ***<0.001, ****<0.0001.

The results of the current study showed a significant increase in blood glucose levels (mg/dL) in diabetic rats when compared to normal control rats. The results also showed that ingesting kernels or caffeine caused a significant decrease in blood glucose levels compared to the diabetic group, and the effect of their combination showed a more substantial impact on lowering blood glucose levels in diabetic rats. The changes in blood glucose levels in different experimental groups are presented in Figure 1A.

### 3.2 Kernel increased insulin levels in diabetic rats more efficiently than caffeine

The induced diabetic rats exhibited a significant decrease in insulin levels (mIU/L) compared to normal control rats. The administration of kernel alone and caffeine alone increased insulin levels; however, kernel showed a better result than caffeine when compared with the diabetic group. The addition of caffeine to the kernel displayed a more significant effect on increasing insulin levels in diabetic rats. The changes in insulin levels in different experimental groups are shown in Figure 1B.

### 3.3 Addition of caffeine to kernel had the most effect in decreasing hemoglobin A1c

Hemoglobin A1c (%) is the percentage of glucose-coated hemoglobin in red blood cells. It shows the average blood sugar level over the length of the experiment. There was a statistically significant difference in the average HbA1c% between the five groups of mice. The highest value of HbA1c% was found in the induced diabetic rats, while the greatest decrease compared to the normal group was found in type 2 diabetic rats treated with apricot kernels plus caffeine. The changes in hemoglobin A1c percentage in different experimental groups are shown in Figure 1C.

### 3.4 Kernel and caffeine decreased body weight in diabetic rats

The body weights of rats at the end of the experiment are shown in Figure 1D and Table 1. There was a statistically significant difference in the mean body weight at the end of the experiment between the five groups, and after conducting Tukey’s *post hoc* test, the highest rate of decrease in body weight was in the induced diabetic group, while the lowest decrease in weight was in the type 2 diabetic rats treated with apricot kernels plus caffeine.

**TABLE 1:** Histological pattern of the Liver.

	Portal tract			CV	Hepatocyte	Blood sinusoids	Intra-lobular inflammatory infiltrate
	PV	Inflammatory infiltrate	Edema				
Group 1	0	0	0	0	0	0	0
Group 2	++	0	+	++	++	0	0
Group 3	++	0	+	0	0	0	0
Group 4	+	+	0	+	++	0	+
Group 5	+	0	0	++	+	+	0

❖ Portal tract.

⁃ Portal vein (PV): 0: average; +: mildly dilated/congested; ++: markedly dilated/congested.

⁃ Inflammatory infiltrate: 0: no; +: mild; ++: moderate/marked.

⁃ Edema: 0: no; +: present.

❖ Central vein (CV): 0: average; +: dilated/congested; ++: markedly dilated/detached lining.

❖ Hepatocytes: 0: average; +: hydropic change; ++: apoptotic/necrotic.

❖ Blood sinusoids: 0: average; +: mildly dilated/congested; ++: markedly dilated/congested.

❖ Intra-lobular inflammatory infiltrate: 0: no; +: mild; ++: moderate/marked.

### 3.5 Improvement of lipid profile in diabetics after administration of kernel

**FIGURE 2:**
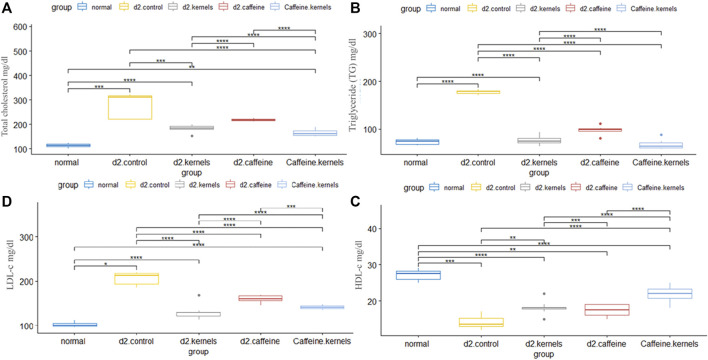
Apricot kernel and caffeine had a positive effect on lipid profile of diabetic rats. **(A)** Total cholesterol level in diabetic rats after different treatments. **(B)** Triglyceride (TG) level in different groups. **(C)** High-density lipoprotein cholesterol (HDL-C). **(D)** Low-density lipoprotein cholesterol (LDL-C). Healthy control rats (normal), control diabetic rats (d2.control), diabetic rats treated with apricot kernel (d2.kernels), diabetic rats treated with caffeine (d2.caffeine), and diabetic rats treated with both caffeine and apricot kernel (Caffeine.kernels). One-way ANOVA and *post hoc* test, *p*-value* <0.05, **<0.01, ***<0.001, ****<0.0001.

In T2DM, hyperglycemia is accompanied by an abnormal lipid profile characterized by imbalances in lipoproteins and cholesterol levels. In the current study, there was a significant increase in the level of TC, triglycerides (TG), and low-density lipoprotein cholesterol (LDL-C). In contrast, high-density lipoprotein cholesterol (HDL-C) was decreased in the diabetic control group in comparison with normal control rats. Kernel or/and caffeine administration showed a significant decrease in the levels of TC, TG, and LDL-C in type 2 diabetic rats. There was also a significant increase in HDL-C in type 2 diabetic rats treated with apricot kernels plus caffeine. The changes in lipid profiles in different experimental groups are shown in Figure 2.

### 3.6 Administration of both kernel and caffeine enhanced antioxidant profile in diabetic rats

**FIGURE 3:**
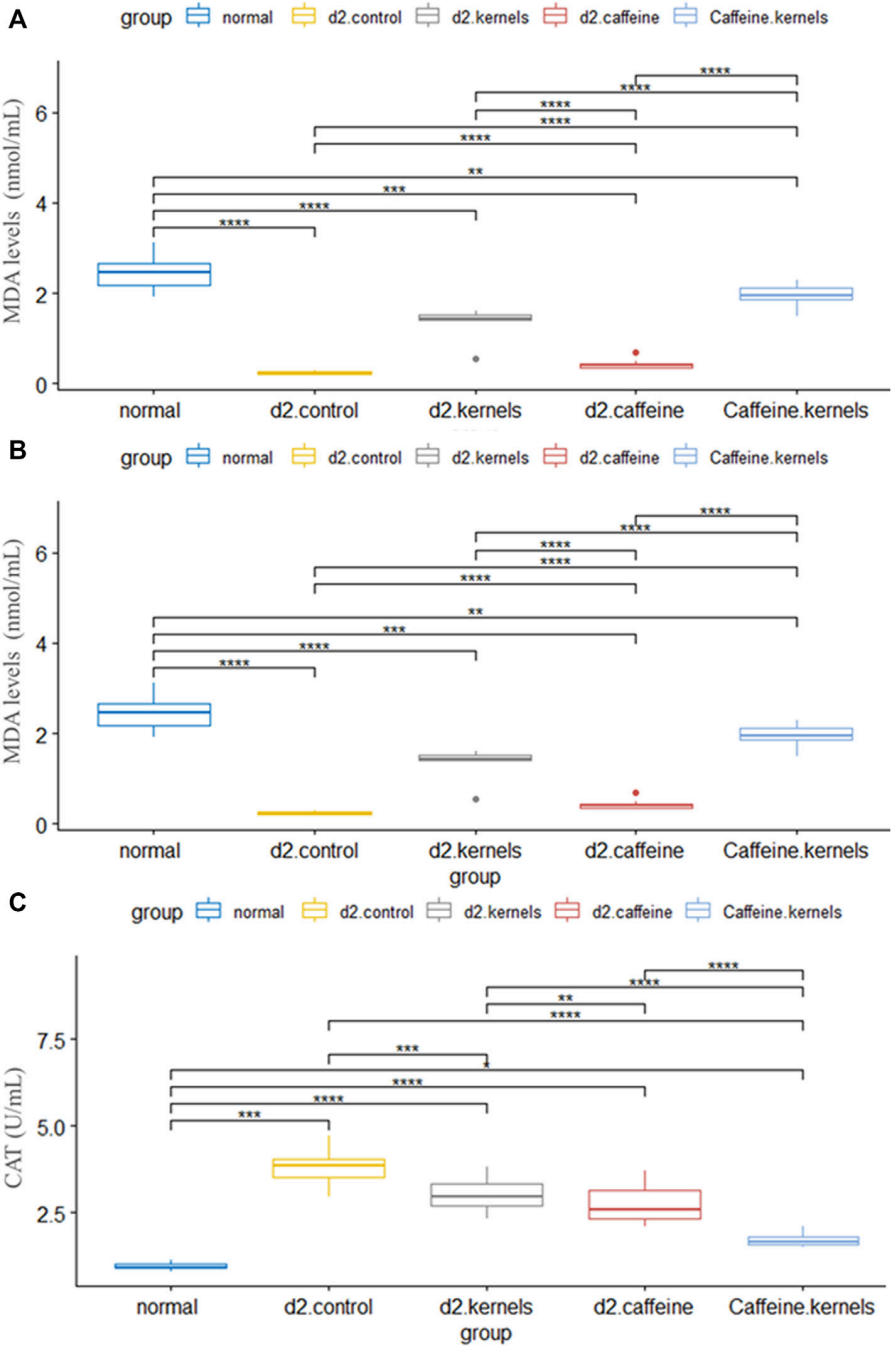
Antioxidant profile in diabetic rats improved after the administration of apricot kernel and caffeine. **(A)** Malondialdehyde (MDA) level in diabetic rats after different treatments. **(B)** Glutathione peroxidase activity (GP_x_). **(C)** Catalase activity (CAT) level in diabetic rats after different treatments. Healthy control rats (normal), control diabetic rats (d2.control), diabetic rats treated with apricot kernel (d2.kernels), diabetic rats treated with caffeine (d2.caffeine), and diabetic rats treated with both caffeine and apricot kernel (Caffeine.kernels). One-way ANOVA and *post hoc* test, *p*-value* <0.05, **<0.01, ***<0.001, ****<0.0001.

Antioxidants play a crucial role in maintaining overall health and may have specific benefits for managing diabetes type 2. Malondialdehyde (MDA), glutathione peroxidase activity GPX (U/mL), and catalase activity CAT (U/mL) are important markers of lipid peroxidation. There was a statistically significant difference in the mean MDA, GPX, and CAT between the five groups, and after performing Tukey’s *post hoc* test, the significance among all groups was significant (*p* < 0.001), except it was not significant between the caffeine and the control group. In contrast, the type 2 diabetic rats treated with apricot kernels plus caffeine had the highest increase in MDA and GPX, while there was a significant decrease in the level of level CAT, approaching the normal level. The changes in antioxidant levels in different experimental groups are shown in Figure 3.

### 3.7 Histopathological analysis

#### 3.7.1 Liver tissue

**FIGURE 4:**
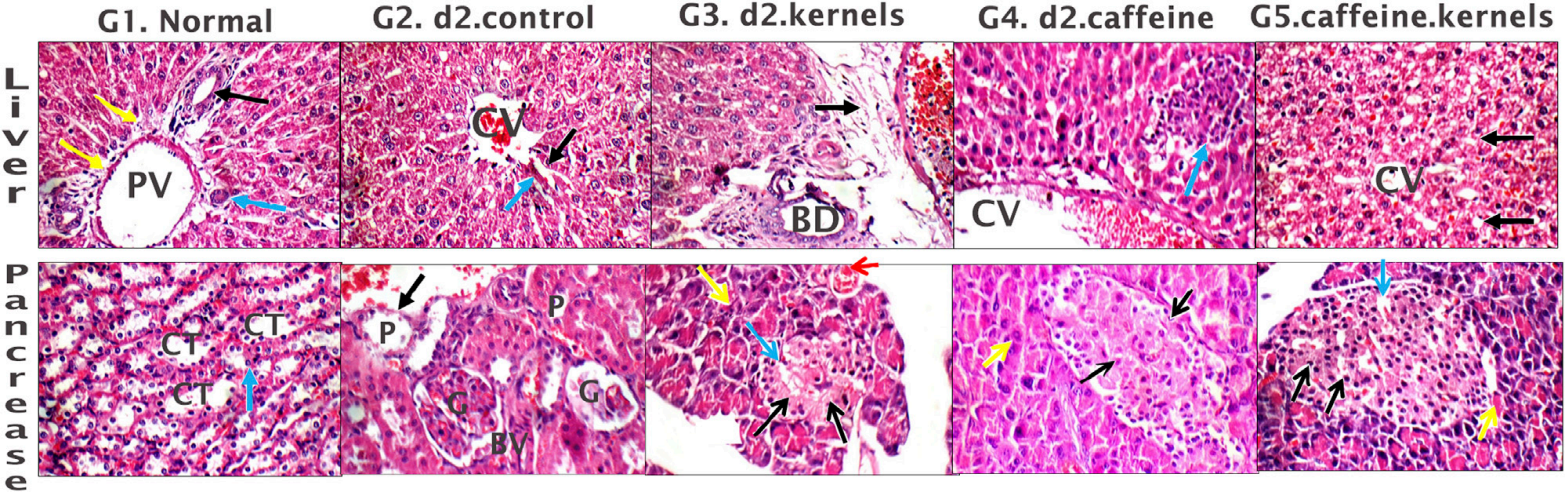
Histological sections of the liver and pancreas. Liver tissue: G1. Control group displays average portal tract size with average portal vein (PV) size, average bile ducts (black arrow), average hepatic artery (blue arrow), and average hepatocytes in peri-portal area (yellow arrow). G2. Diabetic rats treated with normal saline group shows liver with mildly edematous portal tract (black arrow), markedly dilated congested PV, dilated congested central veins (CV), and average hepatocytes (blue arrow). G3. Diabetic rats treated with apricot kernels group exhibits markedly edematous portal tract (black arrow) with markedly dilated congested PV, average bile ducts (BD), and average hepatocytes in peri-portal area (blue arrows). G4. Diabetic rats treated with caffeine group showed mildly dilated congested CV with scattered apoptotic hepatocytes in peri-venular area (black arrow), and intra-lobular inflammatory infiltrate (blue arrow). G5. Diabetic rats treated with apricot kernels plus caffeine group displays average CV with mild micro-vesicular steatosis of hepatocytes in peri-venular area (black arrow). Pancreatic tissue: G1. Control group displays predominating beta cells with pale blue cytoplasm (black arrows) and less frequently alpha cells with pink cytoplasm (blue arrows) separated by average thin-walled blood capillaries (red arrow), and average exocrine areas (yellow arrow). G2. Diabetic rats treated with normal saline group displays hypocellular islets with scattered apoptotic beta cells (black arrow) and mildly congested intervening blood capillaries (blue arrow), average exocrine areas (yellow arrows), and average ducts (red arrow). G3. Diabetic rats treated with apricot kernels group exhibits hypocellular islets with scattered apoptotic beta cells (black arrows) and mildly dilated intervening blood capillaries (blue arrow), average exocrine areas (yellow arrows), and mildly congested interstitial blood vessels (red arrow). G4. Diabetic rats treated with caffeine group show hypocellular islets with scattered apoptotic beta cells (black arrow) and mildly dilated intervening blood capillaries (blue arrow), and average exocrine areas (yellow arrow). G5. Diabetic rats treated with apricot kernels plus caffeine group displays average islets with predominating beta cells (black arrows), mildly dilated congested intervening blood capillaries (blue arrow), and average exocrine areas (yellow arrow) (H&E X 400).

The liver of the normal control showed average portal tracts with average portal veins, average bile ducts, and average hepatocytes in the peri-portal area, and average central veins with average hepatocytes arranged in single-cell cords with average intervening blood sinusoids (Figure 4, G1). However, in the induced diabetes group, mildly edematous portal areas with markedly dilated congested portal veins, markedly dilated central venules with detached lining, and scattered hepatocytes in the peri-portal and peri-portal regions showed pathological changes caused by the induction of diabetes in rats; in turn, these changes reduced the liver’s consumption of glucose, contributing greatly to high blood glucose levels and disturbances in lipid profile and antioxidants as described above (Figure 4, G2). In diabetic rats treated with apricot kernels, the liver showed markedly edematous portal spaces with markedly dilated congested portal venules, intermediate central venules, and intermediate hepatocytes in the peri-portal and peri-venous areas (Figure 4, G3). In diabetic rats treated with caffeine, the liver showed average portal tracts with mild portal inflammatory infiltrate and mildly dilated congested portal veins, mildly dilated congested central veins, and scattered apoptotic hepatocytes in a peri-venular area with intra-lobular inflammatory infiltrate (Figure 4, G4). In diabetic rats treated with apricot kernels plus caffeine, the liver showed average portal tracts with mildly congested portal veins, markedly dilated central veins with mildly congested blood sinusoids, a hydropic change of hepatocytes, and mild micro-vesicular steatosis in the peri-venular area. This necessarily means an improvement in the condition of the liver cells compared to the diabetic control group, as well as an improvement in the rate of glucose consumption by the liver cells, which led to a noticeable decrease in the level of glucose in the blood and an improvement in the lipid profile and antioxidants as described above (Figure 4, G5). The changes in histological structures of different experimental groups are shown in Figure 4 and Table 1.

#### 3.7.2 Pancreas tissue

In the normal control, the pancreas showed average-sized pale-staining islets of Langerhans composed of predominating beta cells with pale blue cytoplasm, and less frequently alpha cells with pink cytoplasm separated by average thin-walled blood capillaries, average exocrine areas, and average ducts (Figure 4, G1). In the induced diabetes group, the pancreas showed small-sized islets of Langerhans with scattered apoptotic beta cells, mildly dilated overlapping capillaries, moderately dilated interstitial blood vessels, intermediate exocrine zones, and intermediate ducts. This severe inflammation of the pancreatic cells led, of course, to decreased insulin secretion by beta cells and thus a significant increase in blood glucose levels (Figure 4, G2). In diabetic rats treated with apricot kernels**,** the pancreas showed small-sized hypocellular islets of Langerhans with scattered apoptotic beta cells and mildly dilated congested intervening blood capillaries, mildly congested interstitial blood vessels, average exocrine areas, and average ducts (Figure 4, G3). In diabetic rats treated with caffeine, the pancreas showed average-sized hypocellular islets of Langerhans with scattered apoptotic beta cells and mildly dilated intervening blood capillaries, average exocrine areas, and average ducts (Figure 4, G4). In diabetic rats treated with apricot kernels plus caffeine, the pancreas showed moderately sized islets of Langerhans with predominant beta cells, mildly dilated interstitial capillaries, mildly dilated interstitial blood vessels, moderate exocrine zones, and mesenchymal ducts. This means a significant improvement in the condition of pancreatic cells when compared to the diabetic control group. This improvement in the morphology of the cells led to a clear improvement in the level of glucose in the blood (Figure 4, G5). The changes in histological structures of different experimental groups were shown in Figure 4 and Table 2.

**TABLE 2:** Histological pattern of the pancreas.

	Pancreatic islets				Ducts	Exocrine area	BV
	Islet size	Cellularity	Beta cells	Capillaries			
Group 1	0	0	0	0	0	0	0
Group 2	+	+	+	+	0	0	+
Group 3	+	+	+	+	0	0	+
Group 4	0	+	+	+	0	0	0
Group 5	0	0	0	+	0	0	+

❖ Islet size: 0: average; +: small; ++: atrophied.

❖ Cellularity: 0: average; +: hypocellular; ++: acellular.

❖ Beta cells: 0: average; +: few/apoptotic; ++: necrotic/absent.

❖ Capillaries: 0: average; +: mildly dilated; ++: markedly dilated/congested.

❖ Ducts: 0: average; +: dilated; ++: atrophied.

❖ Exocrine area: 0: average acini; +: small acini; ++: atrophied acini.

Interstitial BV: 0: average; +: mildly dilated; ++: markedly dilated/congested.

## 4 Discussion

In this study, T2DM was induced in male albino rats fed with a high-fat diet for 3 weeks, followed by a low dose of streptozotocin (Nath et al., 2017). Low streptozotocin doses partially damage pancreatic beta cells and cause the breakage of DNA strands (Szkudelski, 2001). The alkylation of DNA is followed by cell death, and then consequently, an increase in blood sugar levels. Both a high-fat diet and streptozotocin mimic the human syndrome T2DM (Rais et al., 2022). Apricot kernel oil contains some biologically active substances, such as β-carotene, phenolic compounds, campesterol, stigmasterol, sitosterol, and provitamin A (Fratianni et al., 2018). The presence of tocopherols, vitamin C, saponins, oleic acid, and amygdalin in apricot kernel boosts its antioxidant activity and anti-lipemic capacity (Dawod and Ahmed, 2021). Specifically, 100 mg/kg of amygdalin can increase gene expression of glutathione peroxidase and superoxide dismutase in the hepatic tissue of mice (Dawod and Ahmed, 2021).

The reduction of blood glucose levels is the primary therapeutic goal for controlling diabetes (Ceriello et al., 2022). In the present study, the blood glucose level of diabetic rats was significantly decreased when treated with apricot kernels, and this effect was synergized when the kernel was combined with caffeine. The reduction of blood glucose may be either due to the increased level of plasma insulin in diabetic rats, which may influence the stimulation of pancreatic insulin secretion from beta cells in islets of Langerhans, or due to the enhanced transport of blood glucose to peripheral tissue. The mass loss and functionality of pancreatic beta cells have been the major concerns in the pathogenesis of T2DM (Dawood et al., 2023). Functional improvement by increasing the expression of genes linked to beta cell function and insulin biogenesis is one of the mechanisms of the apricot kernel in boosting insulin secretion (Anwar et al., 2018) and improving insulin sensitivity, which is an important therapeutic approach to treating T2DM (Blahova et al., 2021). It could also improve glucose tolerance, augment energy expenditure, and reduce adiposity (Szkudelski, 2001); apricot kernels can be considered a hypoglycemic agent, protecting beta cells, improving their function ability, and acting as hepatoprotective agents (Peng et al., 2023).

Hyperglycemia is accompanied by dyslipidemia, characterized by an increase in TC, LDL, and TG and a fall in HDL. This altered serum lipid profile was reversed to normal after treatment with metformin, as mentioned by Mullugeta et al. (2012). In this study, there was an increase in plasma TG, TC, and LDL-C fractions along with a decreased HDL-C level in the diabetic control group. The HDL-C levels in diabetic and non-diabetic rats fed with apricot kernels were found to be higher compared to the diabetic control group, an indication of a beneficial effect of the apricot kernels, while TG, TC, and LDL levels were decreased in both diabetic and non-diabetic rats fed with apricot kernels. Similarly, consumption of apricot kernels daily had a positive effect on lowering plasma TC and LDL-C in patients with dyslipidemia without a significant effect on plasma cholesterol levels (Zibaeenezhad et al., 2017; Kopčeková et al., 2021; Kopčeková et al., 2022). Furthermore, it reduced hepatic lipid synthesis by decreasing the activity of lipogenic enzymes and increasing hepatic lipolytic enzymes, consequently lowering plasma TG (Sun et al., 2020; Dinda and Dinda, 2022; Kopčeková et al., 2022). The possible mechanism of the anti-hyperlipidemic effect of caffeine might include the changed activity of cholesterol biosynthesis enzymes and/or the changed level of lipolysis, which are under the control of insulin, as mentioned by Zhang et al. (2013). It is reported that caffeine treatment could decrease the capacity of LDL to carry free cholesterol to various tissues without affecting the capacity of HDL to carry cholesterol back to the liver in rats (Ontawong et al., 2019). In addition, apricot kernel treatment can correct hyperlipidemia in rats, as reported by Kutlu et al. (2009). Our results indicated that the lipid-lowering effect could be an indirect consequence of amelioration of insulin resistance or a direct hypolipidemic effect mediated through other mechanisms.

Apricot mostly contains phytochemicals that reduce the risk of free radicals, which cause oxidative damage in living cells and common degenerative disorders such as cancer and cardiovascular diseases; the major anti-inflammatory compounds are acetylcholinesterase (Vahedi-Mazdabadi et al., 2020). Cyclooxygenase, interleukin 6, prostaglandin, Toll-like receptors, and tumor necrosis factor alpha (Huang et al., 2011). The antioxidant potential of apricot has been repeatedly investigated through different *in vitro* systems by measuring its ability to reduce free radicals and comparing it with standard reference compounds (Arora et al., 2017). Tissue MDA content, the final product of lipid breakdown caused by oxidative stress, is an important indicator of free radical-induced lipid peroxidation (Tsikas, 2017). In our study, there was a significant decrease in MDA in diabetic rats treated with apricot and caffeine, which indicates the positive effect of apricot kernels as an antioxidant. MDA is one of the final products of polyunsaturated fatty acid peroxidation in the cells (Tsikas, 2017). An increase in free radicals causes overproduction of MDA. Therefore, it is commonly known as a marker of oxidative stress and the antioxidant status in patients (Demirci-Cekic et al., 2022). There was a significant increase in GPx in diabetic rats treated with apricot and caffeine, which are the most important selenoenzymes due to their role in a diverse range of biological functions, including the detoxification of H_2_O_2_ and hydroperoxides (Lima-Cabello et al., 2023). The catalase activity was significantly increased in diabetic rats treated with apricot and caffeine. Measurements of catalase activity function as a good evaluating tool for assessing the liver’s capacity to attenuate the inclination toward oxidative stress (Sousa et al., 2020).

Caffeine had a similar effect to kernel on blood glucose level, HDL-C, HbA1c, and GPX; conversely, the kernel has a better effect on other parameters such as lipid profile and insulin. Caffeine did not exceed kernel in any tested parameter; however, the addition of caffeine to kernel improved its effect in all tested parameters.

In the study of Ramli et al. (2021), caffiene was shown to reduce the risk of metabolic syndrome. It was found to reduce waist circumference, triglyceride levels, low-density lipoprotein-cholesterol levels, and blood pressure. In a systematic review of the effect of caffeine therapy on cardiometabolic markers in rat models of the metabolic syndrome, out of 228 studies retrieved from the search, caffeine was found to favorably reduce obesity and insulin resistance in the rat model of the metabolic syndrome (Alhadi et al., 2023). In the current study, caffeine had a similar effect to kernel on blood glucose level, HDL-C, HbA1c, and GPX; conversely, kernel has a better effect on another parameter, although caffeine yielded no better result than kernel in any parameter. The addition of caffeine to kernel improved its effect on all tested parameters. The study of caffeine and kernel needs more advanced and deeper research.

In conclusion, apricot kernel helped reduce blood glucose levels and lipid content and increase plasma insulin, in addition to its antioxidant effect. The addition of caffeine augments its beneficial properties. The apricot kernel has excellent therapeutic properties and can be considered a potential ingredient in many drugs and food supplements due to its cost effective and ecofriendly nature. Moreover, further work is necessary to elucidate in detail the mechanism of action of the apricot kernel at the cellular and molecular levels.

## Data Availability

The original contributions presented in the study are included in the article/Supplementary Material; further inquiries can be directed to the corresponding author.
